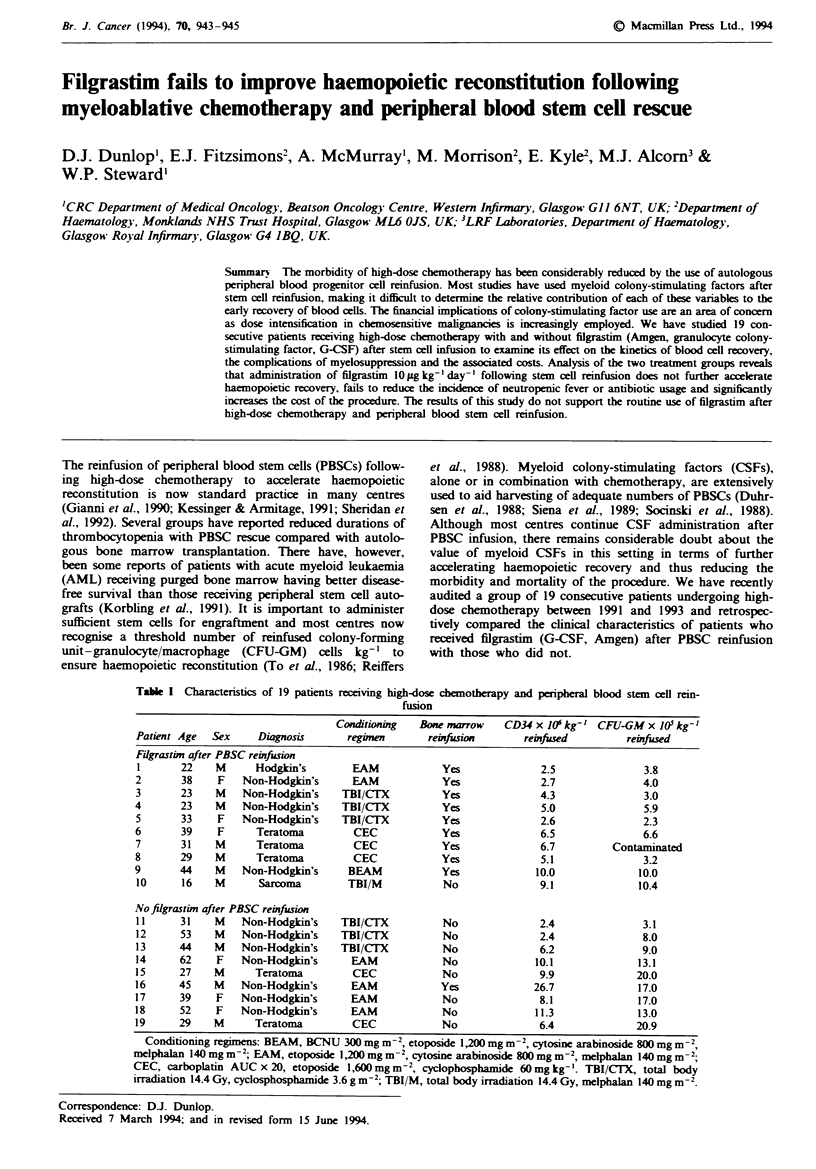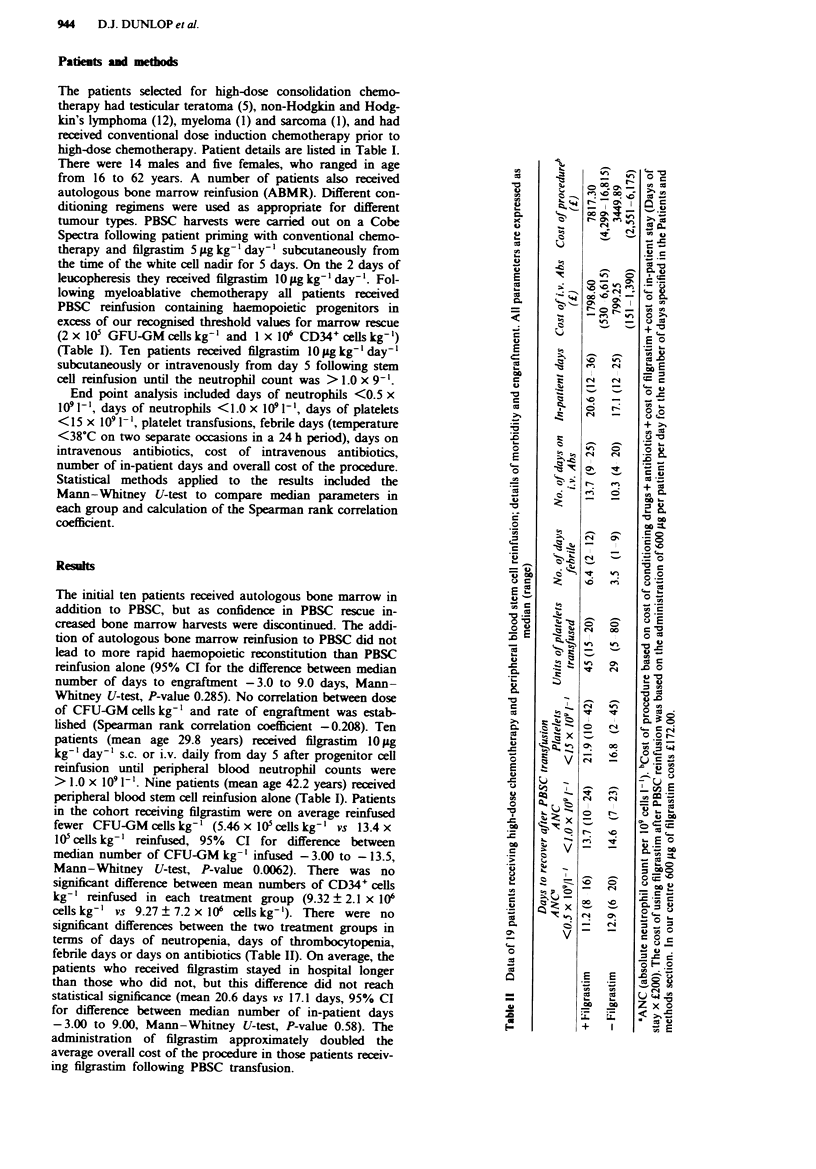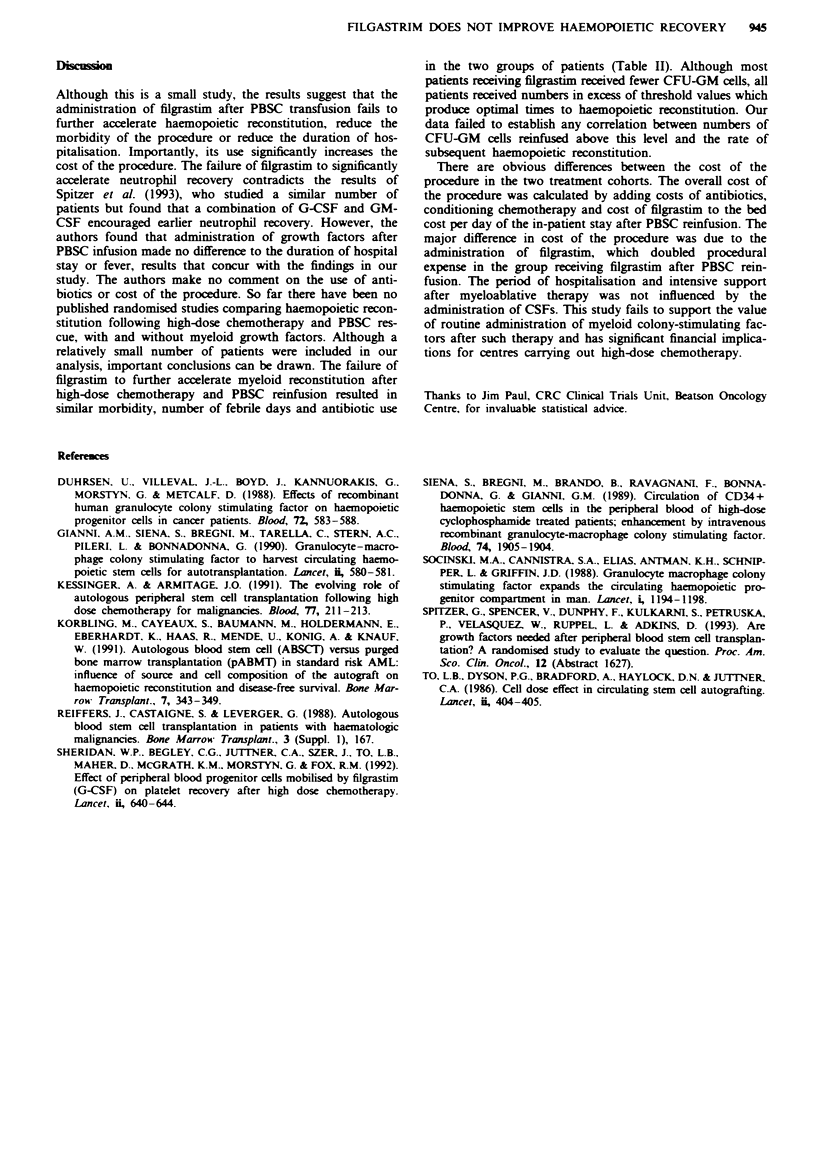# Filgrastim fails to improve haemopoietic reconstitution following myeloablative chemotherapy and peripheral blood stem cell rescue.

**DOI:** 10.1038/bjc.1994.425

**Published:** 1994-11

**Authors:** D. J. Dunlop, E. J. Fitzsimons, A. McMurray, M. Morrison, E. Kyle, M. J. Alcorn, W. P. Steward

**Affiliations:** CRC Department of Medical Oncology, Beatson Oncology Centre, Western Infirmary, Glasgow, UK.

## Abstract

The morbidity of high-dose chemotherapy has been considerably reduced by the use of autologous peripheral blood progenitor cell reinfusion. Most studies have used myeloid colony-stimulating factors after stem cell reinfusion, making it difficult to determine the relative contribution of each of these variables to the early recovery of blood cells. The financial implications of colony-stimulating factor use are an area of concern as dose intensification in chemosensitive malignancies is increasingly employed. We have studied 19 consecutive patients receiving high-dose chemotherapy with and without filgrastim (Amgen, granulocyte colony-stimulating factor, G-CSF) after stem cell infusion to examine its effect on the kinetics of blood cell recovery, the complications of myelosuppression and the associated costs. Analysis of the two treatment groups reveals that administration of filgrastim 10 micrograms kg-1 day-1 following stem cell reinfusion does not further accelerate haemopoietic recovery, fails to reduce the incidence of neutropenic fever or antibiotic usage and significantly increases the cost of the procedure. The results of this study do not support the routine use of filgrastim after high-dose chemotherapy and peripheral blood stem cell reinfusion.


Br. J. Cancer (1994), 70, 943-945 © Macmillan Press Ltd., 1994

### Filgrastim fails to improve haemopoietic reconstitution following myeloablative chemotherapy and peripheral blood stem cell rescue

D.J. Dunlop[1], E.J. Fitzsimons[2], A. McMurray[1], M. Morrison[2], E. Kyle[2], M.J. Alcorn[3] & W.P. Steward[1]

[1]*CRC Department of Medical Oncology, Beatson Oncology Centre, Western Infirmary, Glasgow G11 6NT, UK;* [2]*Department of Haematology, Monklands NHS Trust Hospital, Glasgow ML6 0JS, UK;* [3]*LRF Laboratories, Department of Haematology, Glasgow Royal Infirmary, Glasgow G4 1BQ, UK.*

**Summary** The morbidity of high-dose chemotherapy has been considerably reduced by the use of autologous peripheral blood progenitor cell reinfusion. Most studies have used myeloid colony-stimulating factors after stem cell reinfusion, making it difficult to determine the relative contribution of each of these variables to the early recovery of blood cells. The financial implications of colony-stimulating factor use are an area of concern as dose intensification in chemosensitive malignancies is increasingly employed. We have studied 19 consecutive patients receiving high-dose chemotherapy with and without filgrastim (Amgen, granulocyte colony-stimulating factor, G-CSF) after stem cell infusion to examine its effect on the kinetics of blood cell recovery, the complications of myelosuppression and the associated costs. Analysis of the two treatment groups reveals that administration of filgrastim 10 $\mu g\ kg^{-1}\ day^{-1}$ following stem cell reinfusion does not further accelerate haemopoietic recovery, fails to reduce the incidence of neutropenic fever or antibiotic usage and significantly increases the cost of the procedure. The results of this study do not support the routine use of filgrastim after high-dose chemotherapy and peripheral blood stem cell reinfusion.

The reinfusion of peripheral blood stem cells (PBSCs) following high-dose chemotherapy to accelerate haemopoietic reconstitution is now standard practice in many centres (Gianni *et al.*, 1990; Kessinger & Armitage, 1991; Sheridan *et al.*, 1992). Several groups have reported reduced durations of thrombocytopenia with PBSC rescue compared with autologous bone marrow transplantation. There have, however, been some reports of patients with acute myeloid leukaemia (AML) receiving purged bone marrow having better disease-free survival than those receiving peripheral stem cell autografts (Korbling *et al.*, 1991). It is important to administer sufficient stem cells for engraftment and most centres now recognise a threshold number of reinfused colony-forming unit-granulocyte/macrophage (CFU-GM) cells $kg^{-1}$ to ensure haemopoietic reconstitution (To *et al.*, 1986; Reiffers *et al.*, 1988). Myeloid colony-stimulating factors (CSFs), alone or in combination with chemotherapy, are extensively used to aid harvesting of adequate numbers of PBSCs (Duhrsen *et al.*, 1988; Siena *et al.*, 1989; Socinski *et al.*, 1988). Although most centres continue CSF administration after PBSC infusion, there remains considerable doubt about the value of myeloid CSFs in this setting in terms of further accelerating haemopoietic recovery and thus reducing the morbidity and mortality of the procedure. We have recently audited a group of 19 consecutive patients undergoing high-dose chemotherapy between 1991 and 1993 and retrospectively compared the clinical characteristics of patients who received filgrastim (G-CSF, Amgen) after PBSC reinfusion with those who did not.

**Table I** Characteristics of 19 patients receiving high-dose chemotherapy and peripheral blood stem cell reinfusion

| *Patient* | *Age* | *Sex* | *Diagnosis* | *Conditioning regimen* | *Bone marrow reinfusion* | *CD34* × $10^6\ kg^{-1}$ *reinfused* | *CFU-GM* × $10^5\ kg^{-1}$ *reinfused* |
|---|---|---|---|---|---|---|---|
| *Filgrastim after PBSC reinfusion* | | | | | | | |
| 1 | 22 | M | Hodgkin's | EAM | Yes | 2.5 | 3.8 |
| 2 | 38 | F | Non-Hodgkin's | EAM | Yes | 2.7 | 4.0 |
| 3 | 23 | M | Non-Hodgkin's | TBI/CTX | Yes | 4.3 | 3.0 |
| 4 | 23 | M | Non-Hodgkin's | TBI/CTX | Yes | 5.0 | 5.9 |
| 5 | 33 | F | Non-Hodgkin's | TBI/CTX | Yes | 2.6 | 2.3 |
| 6 | 39 | F | Teratoma | CEC | Yes | 6.5 | 6.6 |
| 7 | 31 | M | Teratoma | CEC | Yes | 6.7 | Contaminated |
| 8 | 29 | M | Teratoma | CEC | Yes | 5.1 | 3.2 |
| 9 | 44 | M | Non-Hodgkin's | BEAM | Yes | 10.0 | 10.0 |
| 10 | 16 | M | Sarcoma | TBI/M | No | 9.1 | 10.4 |
| *No filgrastim after PBSC reinfusion* | | | | | | | |
| 11 | 31 | M | Non-Hodgkin's | TBI/CTX | No | 2.4 | 3.1 |
| 12 | 53 | M | Non-Hodgkin's | TBI/CTX | No | 2.4 | 8.0 |
| 13 | 44 | M | Non-Hodgkin's | TBI/CTX | No | 6.2 | 9.0 |
| 14 | 62 | F | Non-Hodgkin's | EAM | No | 10.1 | 13.1 |
| 15 | 27 | M | Teratoma | CEC | No | 9.9 | 20.0 |
| 16 | 45 | M | Non-Hodgkin's | EAM | Yes | 26.7 | 17.0 |
| 17 | 39 | F | Non-Hodgkin's | EAM | No | 8.1 | 17.0 |
| 18 | 52 | F | Non-Hodgkin's | EAM | No | 11.3 | 13.0 |
| 19 | 29 | M | Teratoma | CEC | No | 6.4 | 20.9 |

Conditioning regimens: BEAM, BCNU 300 mg $m^{-2}$, etoposide 1,200 mg $m^{-2}$, cytosine arabinoside 800 mg $m^{-2}$, melphalan 140 mg $m^{-2}$; EAM, etoposide 1,200 mg $m^{-2}$, cytosine arabinoside 800 mg $m^{-2}$, melphalan 140 mg $m^{-2}$; CEC, carboplatin AUC × 20, etoposide 1,600 mg $m^{-2}$, cyclophosphamide 60 mg $kg^{-1}$. TBI/CTX, total body irradiation 14.4 Gy, cyclophosphamide 3.6 g $m^{-2}$; TBI/M, total body irradiation 14.4 Gy, melphalan 140 mg $m^{-2}$.

Correspondence: D.J. Dunlop.
Received 7 March 1994; and in revised form 15 June 1994.

### Patients and methods

The patients selected for high-dose consolidation chemotherapy had testicular teratoma (5), non-Hodgkin and Hodgkin's lymphoma (12), myeloma (1) and sarcoma (1), and had received conventional dose induction chemotherapy prior to high-dose chemotherapy. Patient details are listed in Table I. There were 14 males and five females, who ranged in age from 16 to 62 years. A number of patients also received autologous bone marrow reinfusion (ABMR). Different conditioning regimens were used as appropriate for different tumour types. PBSC harvests were carried out on a Cobe Spectra following patient priming with conventional chemotherapy and filgrastim 5 $\mu g\ kg^{-1}\ day^{-1}$ subcutaneously from the time of the white cell nadir for 5 days. On the 2 days of leucopheresis they received filgrastim 10 $\mu g\ kg^{-1}\ day^{-1}$. Following myeloablative chemotherapy all patients received PBSC reinfusion containing haemopoietic progenitors in excess of our recognised threshold values for marrow rescue ($2 \times 10^5$ GFU-GM cells $kg^{-1}$ and $1 \times 10^6$ $CD34^+$ cells $kg^{-1}$) (Table I). Ten patients received filgrastim 10 $\mu g\ kg^{-1}\ day^{-1}$ subcutaneously or intravenously from day 5 following stem cell reinfusion until the neutrophil count was $>1.0 \times 9^{-1}$.

End point analysis included days of neutrophils $<0.5 \times 10^9\ l^{-1}$, days of neutrophils $<1.0 \times 10^9\ l^{-1}$, days of platelets $<15 \times 10^9\ l^{-1}$, platelet transfusions, febrile days (temperature <38°C on two separate occasions in a 24 h period), days on intravenous antibiotics, cost of intravenous antibiotics, number of in-patient days and overall cost of the procedure. Statistical methods applied to the results included the Mann–Whitney *U*-test to compare median parameters in each group and calculation of the Spearman rank correlation coefficient.

### Results

The initial ten patients received autologous bone marrow in addition to PBSC, but as confidence in PBSC rescue increased bone marrow harvests were discontinued. The addition of autologous bone marrow reinfusion to PBSC did not lead to more rapid haemopoietic reconstitution than PBSC reinfusion alone (95% CI for the difference between median number of days to engraftment −3.0 to 9.0 days, Mann–Whitney *U*-test, *P*-value 0.285). No correlation between dose of CFU-GM cells $kg^{-1}$ and rate of engraftment was established (Spearman rank correlation coefficient −0.208). Ten patients (mean age 29.8 years) received filgrastim 10 $\mu g\ kg^{-1}\ day^{-1}$ s.c. or i.v. daily from day 5 after progenitor cell reinfusion until peripheral blood neutrophil counts were $>1.0 \times 10^9\ l^{-1}$. Nine patients (mean age 42.2 years) received peripheral blood stem cell reinfusion alone (Table I). Patients in the cohort receiving filgrastim were on average reinfused fewer CFU-GM cells $kg^{-1}$ ($5.46 \times 10^5$ cells $kg^{-1}$ *vs* $13.4 \times 10^5$ cells $kg^{-1}$ reinfused, 95% CI for difference between median number of CFU-GM $kg^{-1}$ infused −3.00 to −13.5, Mann–Whitney *U*-test, *P*-value 0.0062). There was no significant difference between mean numbers of $CD34^+$ cells $kg^{-1}$ reinfused in each treatment group ($9.32 \pm 2.1 \times 10^6$ cells $kg^{-1}$ *vs* $9.27 \pm 7.2 \times 10^6$ cells $kg^{-1}$). There were no significant differences between the two treatment groups in terms of days of neutropenia, days of thrombocytopenia, febrile days or days on antibiotics (Table II). On average, the patients who received filgrastim stayed in hospital longer than those who did not, but this difference did not reach statistical significance (mean 20.6 days *vs* 17.1 days, 95% CI for difference between median number of in-patient days −3.00 to 9.00, Mann–Whitney *U*-test, *P*-value 0.58). The administration of filgrastim approximately doubled the average overall cost of the procedure in those patients receiving filgrastim following PBSC transfusion.

Although this is a small study, the results suggest that the administration of filgrastim after PBSC transfusion fails to further accelerate haemopoietic reconstitution, reduce the morbidity of the procedure or reduce the duration of hospitalisation. Importantly, its use significantly increases the cost of the procedure. The failure of filgrastim to significantly accelerate neutrophil recovery contradicts the results of Spitzer *et al.* (1993), who studied a similar number of patients but found that a combination of G-CSF and GM-CSF encouraged earlier neutrophil recovery. However, the authors found that administration of growth factors after PBSC infusion made no difference to the duration of hospital stay or fever, results that concur with the findings in our study. The authors make no comment on the use of antibiotics or cost of the procedure. So far there have been no published randomised studies comparing haemopoietic reconstitution following high-dose chemotherapy and PBSC rescue, with and without myeloid growth factors. Although a relatively small number of patients were included in our analysis, important conclusions can be drawn. The failure of filgrastim to further accelerate myeloid reconstitution after high-dose chemotherapy and PBSC reinfusion resulted in similar morbidity, number of febrile days and antibiotic use in the two groups of patients (Table II). Although most patients receiving filgrastim received fewer CFU-GM cells, all patients received numbers in excess of threshold values which produce optimal times to haemopoietic reconstitution. Our data failed to establish any correlation between numbers of CFU-GM cells reinfused above this level and the rate of subsequent haemopoietic reconstitution.

There are obvious differences between the cost of the procedure in the two treatment cohorts. The overall cost of the procedure was calculated by adding costs of antibiotics, conditioning chemotherapy and cost of filgrastim to the bed cost per day of the in-patient stay after PBSC reinfusion. The major difference in cost of the procedure was due to the administration of filgrastim, which doubled procedural expense in the group receiving filgrastim after PBSC reinfusion. The period of hospitalisation and intensive support after myeloablative therapy was not influenced by the administration of CSFs. This study fails to support the value of routine administration of myeloid colony-stimulating factors after such therapy and has significant financial implications for centres carrying out high-dose chemotherapy.

Thanks to Jim Paul, CRC Clinical Trials Unit, Beatson Oncology Centre, for invaluable statistical advice.

### References

DUHRSEN, U., VILLEVAL, J.-L., BOYD, J., KANNUORAKIS, G., MORSTYN, G. & METCALF, D. (1988). Effects of recombinant human granulocyte colony stimulating factor on haemopoietic progenitor cells in cancer patients. *Blood*, **72**, 583–588.

GIANNI, A.M., SIENA, S., BREGNI, M., TARELLA, C., STERN, A.C., PILERI, L. & BONNADONNA, G. (1990). Granulocyte-macrophage colony stimulating factor to harvest circulating haemopoietic stem cells for autotransplantation. *Lancet*, **ii**, 580–581.

KESSINGER, A. & ARMITAGE, J.O. (1991). The evolving role of autologous peripheral stem cell transplantation following high dose chemotherapy for malignancies. *Blood*, **77**, 211–213.

KORBLING, M., CAYEAUX, S., BAUMANN, M., HOLDERMANN, E., EBERHARDT, K., HAAS, R., MENDE, U., KONIG, A. & KNAUF, W. (1991). Autologous blood stem cell (ABSCT) versus purged bone marrow transplantation (pABMT) in standard risk AML: influence of source and cell composition of the autograft on haemopoietic reconstitution and disease-free survival. *Bone Marrow Transplant.*, **7**, 343–349.

REIFFERS, J., CASTAIGNE, S. & LEVERGER, G. (1988). Autologous blood stem cell transplantation in patients with haematologic malignancies. *Bone Marrow Transplant.*, 3 (Suppl. 1), 167.

SHERIDAN, W.P., BEGLEY, C.G., JUTTNER, C.A., SZER, J., TO, L.B., MAHER, D., McGRATH, K.M., MORSTYN, G. & FOX, R.M. (1992). Effect of peripheral blood progenitor cells mobilised by filgrastim (G-CSF) on platelet recovery after high dose chemotherapy. *Lancet*, **ii**, 640–644.

SIENA, S., BREGNI, M., BRANDO, B., RAVAGNANI, F., BONNADONNA, G. & GIANNI, G.M. (1989). Circulation of CD34+ haemopoietic stem cells in the peripheral blood of high-dose cyclophosphamide treated patients; enhancement by intravenous recombinant granulocyte-macrophage colony stimulating factor. *Blood*, **74**, 1905–1904.

SOCINSKI, M.A., CANNISTRA, S.A., ELIAS, ANTMAN, K.H., SCHNIPPER, L. & GRIFFIN, J.D. (1988). Granulocyte macrophage colony stimulating factor expands the circulating haemopoietic progenitor compartment in man. *Lancet*, **i**, 1194–1198.

SPITZER, G., SPENCER, V., DUNPHY, F., KULKARNI, S., PETRUSKA, P., VELASQUEZ, W., RUPPEL, L. & ADKINS, D. (1993). Are growth factors needed after peripheral blood stem cell transplantation? A randomised study to evaluate the question. *Proc. Am. Sco. Clin. Oncol.*, **12** (Abstract 1627).

TO, L.B., DYSON, P.G., BRADFORD, A., HAYLOCK, D.N. & JUTTNER, C.A. (1986). Cell dose effect in circulating stem cell autografting. *Lancet*, **ii**, 404–405.